# A conversation on bringing the Registered Report format to Nature Communications

**DOI:** 10.1038/s41467-022-34325-2

**Published:** 2022-11-28

## Abstract

We recently published our first Registered Report entitled ‘Value-free random exploration is linked to impulsivity’. We believe the format offers many benefits to strengthen hypothesis-driven research and are keen to share our experience with our readers as we open up the format to all fields of research. We interviewed the authors of the manuscript (*Magda Dubois* and *Tobias Hauser*) and one of the reviewers (*Trevor Robbins*) about their experience of the review process. We are editorially committed to take their comments on board to further improve our guidance and to optimally support our future authors.

## The authors’ perspective

**Figure:**
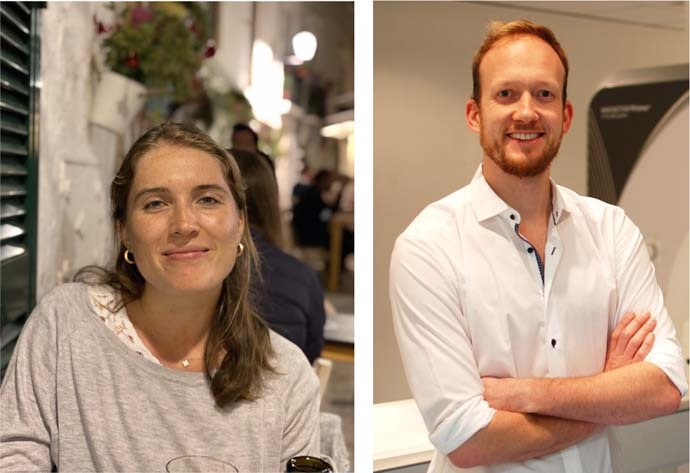
Dr. Magda Dubois and Dr. Tobias Hauser Dr. Magda Dubois and Dr. Tobias Hauser (Dr. Hauser’s photo was taken by Dr. Raphael Köster)

Why did you decide to submit a Registered Report?

We have been following the innovations for a reproducible research culture for several years with great interest. Registered reports, in the format that *Nature Communications* is currently offering, have a great appeal for certain types of projects. Our study was ideally suited for such a Registered Report: we had very clear directed hypotheses, a previously validated task and analysis pipeline, and it was feasible to obtain the preliminary data to prepare a submission.

Moreover, we were aiming to obtain a large and rich data set. Such data sets can invite lengthy and exploratory analyses, which in turn can lead to over-exploration and a failure to rigorously control for multiple testing. Given that we had very clear hypotheses, we decided that a Registered Report was the ideal vehicle to clearly state our directed hypotheses and the more exploratory analyses a priori, and thus be transparent about the findings and their robustness.

This project was thus also an ideal means for us to get to know this novel form of publishing and to gauge its use as a practice for an open and robust science.

What did you perceive to be the main benefits of the Registered Report review process?

There are several clear advantages of Registered Reports. First and foremost, it was very helpful for us to get the reviewers’ feedback from the beginning. This allowed us to implement many suggestions and to make the science more robust. This is a big advantage over the traditional review process.

Second, having laid out (and coded) all the analyses already prior to data collection, made the analysis and writing up stage much quicker and streamlined. Because we already had a structured analysis pipeline, we were able to analyse the data quickly and efficiently.

Lastly, the promise of publication irrespective of the study outcome (or its significance) can make the entire project less stressful and allows one to focus on robustness rather than chasing after significant effects.

What were the challenges you encountered when writing your Stage 1 proposal?

When writing the Stage 1 report, we had to think of all possible analyses that we may want to do and factor in how the data may turn out to be. Essentially, we had to build a complex decision tree and cover as many aspects of it as possible, to plan how to analyse the data, even if the results are different than expected. Given that in many projects, data analysis is often a ‘dialogue’ between the researcher and the data, a Registered Report requires substantially more strategic planning.

A second challenge that we encountered later on was that certain procedures that we had laid out in the Stage 1 report were no longer available because certain registered (analysis) functions had changed or did not exist anymore. Such challenges are probably not pre-emptable in a dynamic environment where open-source software is constantly being further developed.

Thirdly, it was challenging to exactly know how to structure the Stage 1 proposal. In particular, for hypotheses that are less clearly defined or more exploratory, we had to ensure that each was clearly signposted. For example, we had to distinguish between a Stage 1 exploratory analysis (an analysis we have planned but with undirected hypotheses) and a Stage 2 analysis (novel, unplanned hypothesis). The reviewers and editors were extremely helpful in clarifying this and in guiding us in how to present our hypotheses.

Lastly, we struggled with writing a comprehensive introduction and methods section that would stand the test of time and later remain meaningful even if the results deviated substantially from the initial hypotheses. Because these sections are not changeable after Stage 1 acceptance, a lot of consideration should be put into drafting these sections to make sure that the final paper is accessible to the reader.

How did the review process compare to your expectations for this type of submission?

The entire review process took substantially longer than we had expected. We went through two full cycles of reviews, rather than a single one as for a ‘normal’ paper. This means that a Registered Report publication is likely to be slower, and that reviewers play a substantially larger role across the entire process. Having the reviewers’ comments and suggestions early on in the process was of great benefit and helped us pre-empt some limitations that we would not have considered otherwise.

We believe that when authors, editors and reviewers get more used to this form of reviewing, the process will become smoother. For example, we implemented some helpful reviewer suggestions for the introduction in Stage 2, which we then had to revert because they were brought up after Stage 1 acceptance. A very clear guidance for authors and reviewers could help prevent this.

What were the main challenges you encountered during the Registered Report review process?

We have probably underestimated how long a Registered Report project takes. For us the duration was probably the one of a ‘normal’ paper plus an additional full cycle of reviews and revisions. This means that Registered Reports are better suited for researchers who are at the start of a project or position with a relatively flexible timeline.

Moreover, the journal requires a very high power for these Registered Report studies, which could make it difficult to conduct studies with participants that are less readily available, such as with patients or in-person lab experiments; especially if the expected effect sizes are small to moderate. Moreover, it may create inequality because only ‘rich’ labs will be able to afford such large-scale studies.

Is there anything you would do differently the next time?

For future Registered Reports, we will put in place a clearer separation between the different forms and stages of data analysis from the outset. If it is made clear which results belong to which stage/category, it will be easier to adhere to this structure consistently throughout all phases of the process.

We also think that some of the challenges encountered will never be entirely preventable, even with the best thought-through analysis plan. This is because it is difficult to entirely foresee how real data will look before obtaining it, and hence a certain degree of flexibility is needed to adjust to challenges. One example is participant exclusion criteria, which are very difficult to predict even when consulting the literature and one’s own pilot data. This is because large (online) samples may reveal yet different response patterns that were not encountered before.

How do you think the Registered Reports review process at a multidisciplinary journal like *Nature Communications* can be improved?

As this is a fairly new process, we believe that authors, reviewers and editors need some time to adjust to the specific requirements of this new form of publishing. It could be helpful if the reviewers would get more guidance on what the different stages are critical for, and which aspects their review should focus on.

Reviewing a Registered Report is a substantially bigger commitment than reviewing a ‘classic’ paper. If we want reviewers to contribute to such an extensive process, we should perhaps incentivise and acknowledge their contribution accordingly. A substantial input given at the design stage of the study could technically be equal to a traditional co-authorship. At the moment such contributions are not acknowledged at all. Maybe Registered Reports are a good means to fundamentally re-think authorship and contributions to academic research.

*Note from the editors: Nature Communications acknowledges the contribution of reviewers in a peer review information statement on each article, including the names of the reviewers should they wish to be identified. We are exploring additional ways of recognising the work of our referees in the future*.

## A reviewer’s perspective

**Figure:**
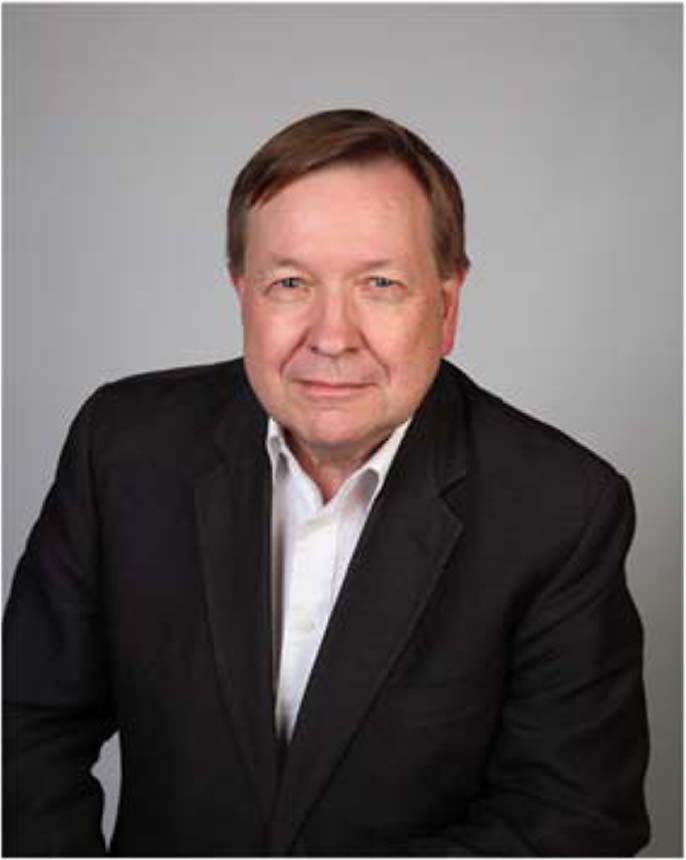
Professor Trevor Robbins Professor Trevor Robbins

Why did you decide to review a Registered Report?

The topic of the manuscript was of considerable scientific interest to me and *Nature Communications* is a good journal (which I hadn’t previously much associated with studies involving human behaviour, probably erroneously).

What did you perceive to be the main benefits of the Registered Report review process?

I suppose for prospective authors it gives them the chance to test the viability of their concept and perhaps benefit from constructive suggestions from expert referees who specialise in such features as experimental design, statistical analysis and computational modelling approaches. I think it’s more difficult for some referees such as myself to do this in the more abstract sense, before data are collected.

How did the review process compare to your previous experiences as a reviewer for primary research? (If you have reviewed for *Nature Communications* before, please comment on how the process differed specifically for *Nature Communications*).

It was frustrating initially not to be able to evaluate actual data. It was not straight-forward to have to focus on the strength of the theory and the study plan without knowledge of the actual findings. I didn’t feel particularly satisfied with my review compared to my more regular efforts.

How did the review process compare to your expectations for reviewing this type of submission?

It felt at times ponderous and somewhat protracted. When evaluating the Stage 2 report with the results, I felt a little constrained in my comments.

What were the main challenges you encountered during the review process?

Wondering whether the manuscript would be accepted for publication regardless of the actual findings. It felt as though the submission was more like a grant application than a scientific manuscript. I was concerned that exploratory data analysis would very much take a back seat in the final version.

Would you agree to review future Registered Reports?

I’m not sure; it would have to depend on the topic.

Would you consider submitting a Registered Report yourself?

Probably not. I think this approach is fine for clinical trial- like and hypothesis-driven projects but not necessarily appropriate for more open-ended scientific research. I can envisage problems arising from possible contrasting requirements of referees, which are acute enough in the normal manuscript reviewing process.

How do you think the Registered Reports review process at a multidisciplinary journal like *Nature Communications* can be improved?

I hope it does not become the norm although if it is popular then by all means make it available. It might be better to have a smaller number of specialised referees offering advice on the initial submission regarding technical aspects, and additional referees then looking at the manuscript when it is submitted in a more complete form.

